# Simultaneous removal of atrazine and copper using polyacrylic acid-functionalized magnetic ordered mesoporous carbon from water: adsorption mechanism

**DOI:** 10.1038/srep43831

**Published:** 2017-03-02

**Authors:** Yaoyu Zhou, Fengfeng Zhang, Lin Tang, Jiachao Zhang, Guangming Zeng, Lin Luo, Yuanyuan Liu, Pei Wang, Bo Peng, Xiaocheng Liu

**Affiliations:** 1 College of Resources and Environment, Hunan Agricultural University, Changsha 410128, China; 2 College of Environmental Science and Engineering, Hunan University, Changsha 410082, China; 3 Key Laboratory of Environmental Biology and Pollution Control, Ministry of Education, Hunan University, Changsha 410082, China; 4 Department of Resources and Environmental Sciences, Changsha University of Science and Technology, Changsha 410114, China

## Abstract

Highly efficient simultaneous removal of atrazine and Cu(II) was accomplished using synthesized polyacrylic acid-functionalized magnetic ordered mesoporous carbon (P-MMC) as compared to magnetic ordered mesoporous carbon (MMC) and ordered mesoporous carbon (OMC). The mutual effects and interactive mechanism of their adsorption onto P-MMC were investigated systematically by binary, preloading and thermodynamic adsorption procedures. In both binary and preloading systems, the adsorption of atrazine was inhibited to some extent by the presence of Cu(II) because of selective recognition and direct competition, but the presence of atrazine had negligible effect on Cu(II) desorption. With the coexistence of humic acid (0–20 mg L^−1^), both atrazine and Cu(II) sorption increased slightly in sole and binary systems. With the concentration of coexisting NaCl increasing from 0 to 100 mM, the adsorption capacity for Cu(II) slightly decreased, but as for atrazine adsorption, it decreased at first, and then increased slightly in sole and binary systems. P-MMC was applied to treat real environmental samples, and the sorption capacities for atrazine and Cu(II) in real samples were all more than 91.47% and 96.43% of those in lab ultrapure water, respectively. Finally, comprehensively considering the relatively good renewability and the superior behavior in the application to real water samples, P-MMC has potential in removal of atrazine, Cu(II) and possibly other persistent organic pollutants from wastewater.

Recalcitrant organic compounds and heavy metals are common in the environment, and give rise to a serious toxicological threat to the ecosystem and human health. For example, atrazine (2-chloro-4-ethylamine-6-isopropylamino-1,3,5-triazine), a model selective triazine herbicide used in agriculture industries, leading to great ecological and human health concern because of its widespread application and persistence^1^,^2^,^3^. Moreover, due to the interference of atrazine endocrine hormone metabolism, some studies suggested that atrazine was considered as a potential carcinogen^4^,^5^. Heavy metals (e.g., copper, lead and cadmium) have been mainly used in agricultural and metal plating/coating industries^6^,^7^. Copper is reported as widespread heavy metals in wastewater and natural environment. Although copper is an essential micronutrient, it can cause copper poisoning at high concentration in humans such as severe headaches, hair loss, hypoglycemia, kidney damage, gastrointestinal problems and even death^8^,^9^.

Additionally, recalcitrant organic compounds and heavy metals commonly coexist in real environments such as agriculture production, bio-treated effluent and dye wastewater, giving rise to more serious damage to the ecosystem and humans due to their combined toxicity and relative mobility^10^,^11^. Consequently, finding effective ways to control these combined pollutants has aroused continuous concern recently.

The traditional methods such as advanced oxidation, chemical precipitation, biological treatment and membrane treatment have been extensively applied to remove recalcitrant organic compounds and/or heavy metals with a positive effect^12^. However, these methods suffer from disadvantages such as high cost, low efficiency, and secondary pollution^13^,^14^. Adsorption, as a green technology, has been widely utilized for the successful removal of recalcitrant organic compounds and/or heavy metals in recent years^15^,^16^,^17^,^18^,^19^. Various adsorbents such as polymeric resins, soil, biosorbents and chitosan have been applied^20^,^21^. For examples, Tao *et al*.^22^ used the natural sediment to remove atrazine and dye compounds simultaneously, and investigated the competitive adsorption between atrazine and dye compounds. Krajnc *et al*.^23^ and Xu *et al*.^21^ applied different polymeric resins to adsorb atrazine with high efficiency, and clarified its removal mechanism; Zhang and co-workers found soil, river and marine sediments could be used to remove tetracycline, and the presence of Cu(II) facilitated tetracycline adsorption on soils and sediments at low pH^24^.

Moreover, searching for new simple, regenerated recycled and inexpensive methods to control recalcitrant organic compounds and heavy metals is also of considerable interest. Alternatively, carbon-based materials (e.g., carbon nanotubes, graphene, biochar and mesoporous carbon) with their controlled pore size distribution, high surface area to volume ratio and manipulatable surface chemistry, overcome many of these intrinsic limitations. Sorption studies using carbon-based nanomaterials was reported as high adsorption capacity, effectiveness and rapid equilibrium rates for the removal of recalcitrant organic compounds and heavy metals^25^. For example, Chen and co-workers illuminated the effect of copper on the sorption of 2,4,6-trichlorophenol and vice versa on multiwalled carbon nanotubes (MWCNTs)^26^. Cao and co-workers evaluated the ability of dairy-manure derived biochar to sorb lead and atrazine. In addition, ordered mesoporous carbons (OMCs) one kind of carbon-based materials, with large surface area and pore volume, unique pore size, and excellent physicochemical and thermal stability are promising candidates in pollutant removal^27^.

In our recent report, we synthesized cobalt nanoparticles-embedded magnetic ordered mesoporous carbon^28^,^29^,^30^ and nitrogen/phosphorus-functionalized magnetic ordered mesoporous carbon with large specific surface area, and sufficient pore regions for the effective removal of recalcitrant organic compounds and heavy metals from wastewater^31^,^32^,^33^,^34^,^35^. These works demonstrated that transition metal elements such as Co and Fe^28^,^29^,^30^ or nonmetal atoms of P and N^31^,^34^ can be used as adsorbent to accomplish primary amorphous ordered mesoporous carbon (OMC) graphitization, strengthening their excellent acid-base stability and hydrophilic properties. For example, the introduction of magnetic nanoparticle of Fe can greatly improve their practical application in separation and reutilization^28^, in addition, the introduction of nonmetal heteroatom of N element, and functionalized polyethylenimine (PEI) or polyacrylic acid (PAA) to equip ordered mesoporous carbon with amino (-NH and -NH_2_) groups, and oxygen containing groups (-OH and -COOH)^35^,^36^,^37^, boosting the chelation with recalcitrant organic compounds and heavy metals.

Based on the above analysis, the purpose of this research was 1) to investigate the removal efficiency of atrazine and/or Cu(II) by polyacrylic acid (PAA)-functionalized magnetic ordered mesoporous carbon (P-MMC), 2) to investigate the mutual effects and inner mechanisms of their adsorption onto P-MMC by sole, preloaded and binary systems, 3) to investigate the influences of water quality parameters on the removal performance of P-MMC including pH value, ionic strength and humic acid.

## Results and Discussion

### Comparisons of adsorption properties for Cu(II) and atrazine onto different mesoporous carbon material. Sole systems for atrazine

**Figure 1:**
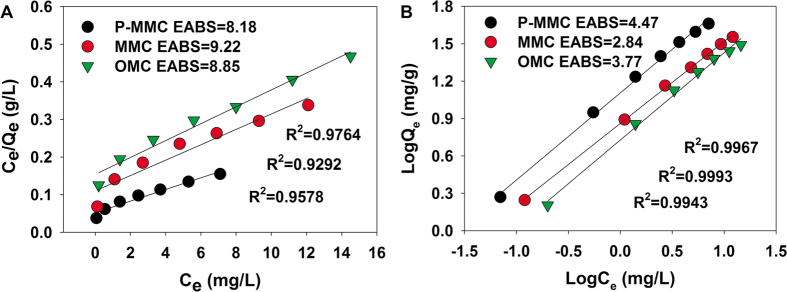
The adsorption isotherms of atrazine by P-MMC, MMC and OMC (initial pH of 5.0, temperature at 303 K).

In order to investigate the effect of magnetic and grafting polyacrylic acid for the removal of atrazine and Cu(II), three mesoporous carbon materials including OMC, MMC and P-MMC were used to compare their adsorption capacities. Freundlich and Langmuir models were both used to describe isotherm data of atrazine. The equations and related parameters of Freundlich and Langmuir models are listed in Text S-2, and the results was presented in Fig. 1 and Table S-1. It was observed that the adsorption capacities for atrazine were in the following order: P-MMC > MMC > OMC. P-MMC exhibited the highest adsorption capacity, because of a larger number of carboxyl and hydroxyl groups in P-MMC may offer additional affinity and more available binding sites for atrazine through hydrogen bonding. The Freundlich model with higher correlation coefficients (*R*^2^) and lower values of the sum of the absolute errors (EABS) could better describe the adsorption isotherms for atrazine than the Langmuir model (Fig. 1), suggesting that pores and some heterogeneity on the surfaces of P-MMC played an important role in atrazine adsorption. The results were consistent with the previous works where the Freundlich isotherm was more suitable than the Langmuir isotherm for the adsorption of atrazine on various adsorbents (Table S-2), such as humic acid and silica gel mixtures^38^, humic acids coated nanoparticles^39^ and magnetic multi-walled carbon nanotube^40^.

In addition, value of 1/n < 1.0 represents an advantageous adsorption condition. Therefore, the Freundlich exponent 1/n gave an indication of the favorability of atrazine adsorption by P-MMC, MMC and OMC. However, according to the *K*_F_ values, P-MMC had the highest adsorption capacity toward atrazine among all tested mesoporous carbon materials.

### Sole systems for Cu(II)

**Figure 2:**
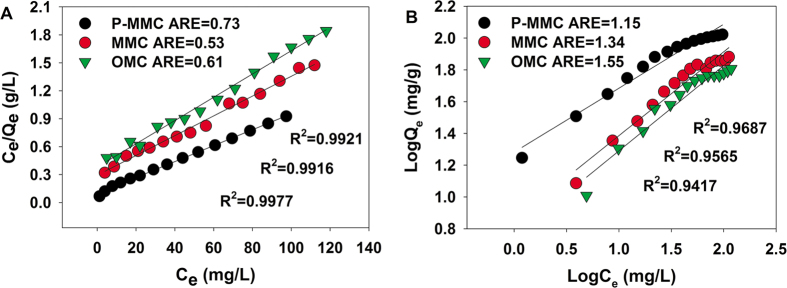
The adsorption isotherms of Cu(II) by P-MMC, MMC and OMC (initial pH of 5.0, temperature at 303 K).

The adsorption isotherms for Cu(II) of these materials are shown in Fig. 2 and Table S-1. It was observed that the adsorption capacity was in the following order: P-MMC > MMC > OMC, P-MMC exhibited the highest adsorption capacity. As shown in Fig. 2, the Langmuir model with higher correlation coefficients and lower values of the average relative error (ARE), gave a better fit to the equilibrium data than the Freundlich model, suggesting that the existence of homogeneous active sites within the adsorbent and the monolayer adsorption of Cu(II) on P-MMC^31^,^37^,^41^. Moreover, P-MMC showed the highest maximum adsorption capacity mainly due to the highest chelating affinity of oxygen containing groups to Cu(II), as reported previously^31^,^42^,^43^,^44^,^45^. In addition, it is noted that P-MMC showed a relatively high sorption capacities for Cu(II) and/or atrazine in comparison with other adsorbents (Table S-3).

### Binary systems

**Figure 3:**
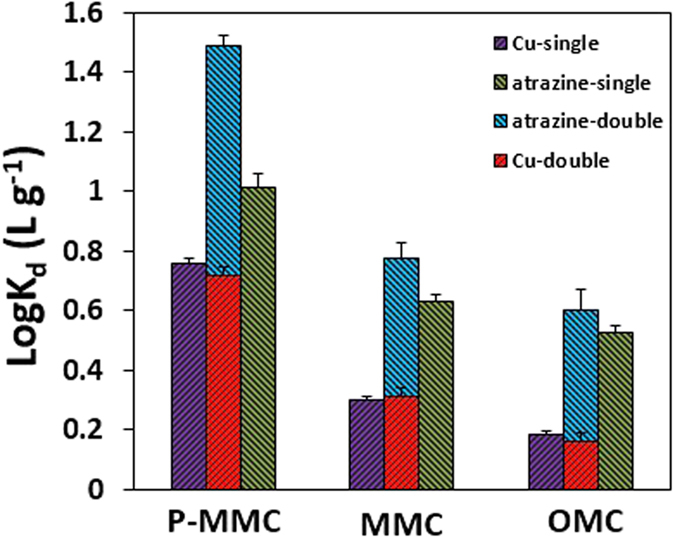
Adsorption amounts for atrazine and Cu(II) onto P-MMC, MMC, and OMC from both single and double systems (initial concentrations of 15 mg·L^−1^ and 30 mg·L^−1^ for atrazine and Cu(II), initial pH of 5.0, temperature at 303 K).

Figure 3 presented the adsorption amounts for atrazine and Cu(II) onto the adsorbents of mesoporous carbon materials from both single and double systems. The distribution coefficients (*K*_d_) are mass weighted partition coefficients of targeted pollutant between the solid and solution phases^35^, the equation and related parameters are listed in Text S-2, Supporting Information. It could be clearly seen that the sorption distribution coefficients (log *K*_d_) of P-MMC was larger than others whether in a single or double systems. Consequently, the superior properties of P-MMC for adsorption of both atrazine and Cu(II) demonstrate the potential for successful simultaneous removal of typical Cu(II) and atrazine, which should be explored further to develop an efficient method.

### Mutual effects upon the adsorption of both atrazine and Cu(II) onto P-MMC. Adsorption isotherms

**Figure 4:**
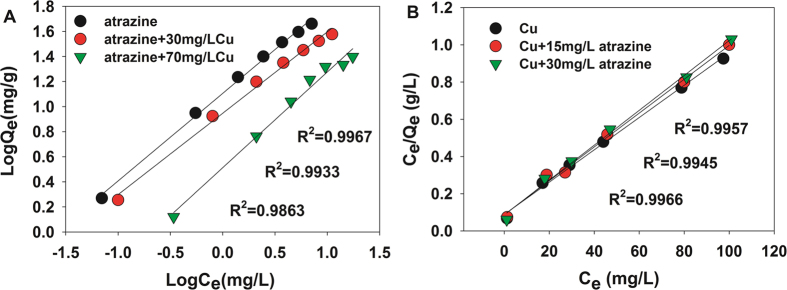
Mutual effect of atrazine and Cu(II) adsorption onto P-MMC; (**A**) Freundlich isotherms for atrazine with interaction to Cu(II) of 0, 30 and 70 mg·L^−1^ tested at 303 K, at an initial pH of 5.0; (**B**) Langmuir isotherms for Cu(II) with interaction to atrazine of 0, 15 and 30 mg·L^−1^ tested at 303 K, at an initial pH of 5.0.

Figure 4A and Fig. S-4A presented the adsorption isotherms for atrazine with or without Cu(II). The amounts of adsorbed atrazine onto P-MMC decreased with the increasing of Cu(II) initial concentration from 0 to 70 mg·L^−1^. As shown in Table S-4, the Freundlich equation fits the experimental data well, indicating that some heterogeneity on the pores or surfaces of P-MMC played an important role in the adsorption of atrazine, and different sites with several adsorption energies were involved^40^,^46^. The Freundlich constant (*K*_F_) of atrazine declined obviously in the presence of Cu(II) with 30 mg·L^−1^ and 70 mg·L^−1^, suggested that Cu(II) had a suppression effect on the atrazine sorption. The suppression impact might be attributed to the direct competition of both adsorbates for similar sites such as hydroxyl and hydroxyl group.

In addition, the adsorption isotherms for Cu(II) from sole and binary systems by P-MMC are compared in Fig. 4B and Fig. S-4B, and the obtained characteristic parameters are tabulated in Table S-4, Supporting Information. The Langmuir model gave a better fit than the Freundlich model. And the impact of atrazine on Cu(II) desorption was almost negligible, suggested that Cu(II) showed a higher affinity toward P-MMC than atrazine.

### Thermodynamic investigations

The thermodynamic equation and parameters are listed in Text-2, Supporting Information. The vant’s Hoff plot (Fig. S-5) is plotted as ln *K*_d_ versus 1/T to calculate ΔH° and ΔS°. The thermodynamic parameters for atrazine and Cu(II) are summarized in Table S-5, Supporting Information. The values of ΔH° for atrazine were all negative, confirming that the adsorption of atrazine onto P-MMC was exothermic^47^. All the ΔH° values were less than 40 kJ mol^−1^ thereby implying that the sorption of atrazine onto P-MMC is mainly a physisorption process^48^,^49^. The positive values of ΔS° implied an increased randomness at the solid−solution interface^50^. The value of ΔG° is negative, indicating the spontaneous nature of the adsorption of atrazine onto P-MMC.

On the contrary, all the values of ΔH° for Cu(II) were positive, indicating that the adsorption of Cu(II) onto P-MMC is endothermic and driven by chemical reaction^10^. And the positive ΔS° suggested that increased randomness at the solid solution interface occurred in the internal structure of the uptake of Cu(II) onto P-MMC. Similarly, the negative value of ΔG° could suggest the feasibility and spontaneity toward Cu(II) adsorption.

### Preloading identification

**Figure 5:**
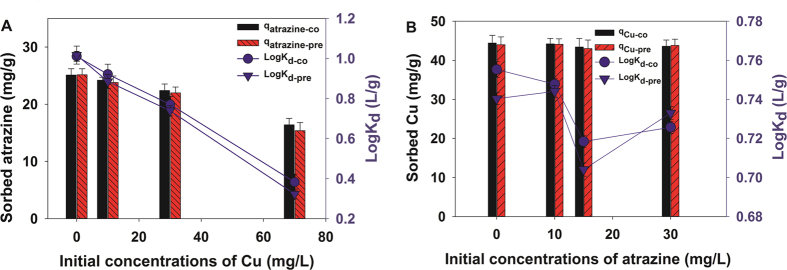
(**A**) The effect of the initial concentration of Cu(II) on atrazine adsorption in preloading and simultaneous adsorption studies. (**B**) The effect of the initial concentration of atrazine on Cu(II) adsorption in preloading and simultaneous adsorption studies (The fixed concentration of atrazine and Cu(II) was 15 mg·L^−1^ and mg·L^−1^, respectively).

**Figure 6:**
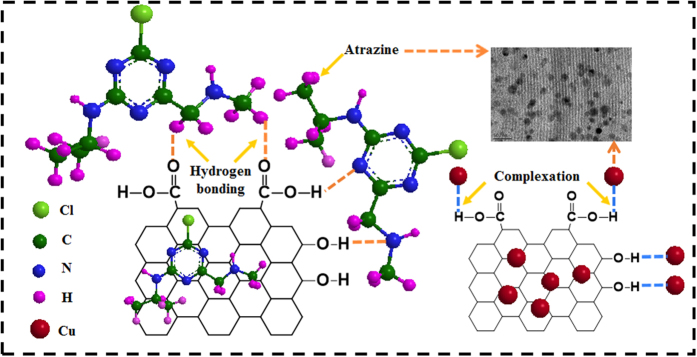
The proposed mechanisms for atrazine and Cu(II) sorption.

To further identify the interaction mechanisms behind the co-adsorption of Cu(II) and atrazine onto P-MMC, a series of preloading equilibrium experiments were also conducted. The sequential adsorption procedures were performed, and the results are shown in Fig. 5. As seen in Fig. 5A, in the simultaneous adsorption studies, the higher Cu(II) concentration, resulting in the smaller values of the sorption distribution coefficients (log *K*_*d-CO*_) and adsorbed atrazine, which suggested that Cu(II) had a suppression effect on the atrazine sorption. In addition, when atrazine was preloaded on P-MMC, the values of the sorption distribution coefficients (log *K*_*d-pre*_) and adsorbed atrazine also decreased with the increase of the initial concentration of Cu(II), suggesting that Cu(II) could compete with atrazine for the same adsorption sites (Fig. 6), and atrazine could be readily displaced by Cu(II), which possessed a stronger binding affinity and accordingly suppressed atrazine adsorption.

Besides, the effects of co-adsorption experiments by fixing Cu(II) concentration but with different concentrations of atrazine, and by preloading P-MMC with Cu(II) followed by exposure to different concentrations of atrazine solution were also investigated, and the results are presented in Fig. 5B. In the co-adsorption studies, varying atrazine concentrations had a relatively smaller suppression effect on the sorption of Cu(II) due to the (log *K*_*d-co*_) and adsorbed Cu(II) show little change. And in the Cu(II)-preloading experiments, the changes of (log *K*_*d-pre*_) and adsorbed Cu(II) are small, suggesting little Cu(II) were desorbed and a stronger binding affinity of Cu(II) on to P-MMC. Actually, the inhibited sorption of recalcitrant organic compounds by heavy metal was previously reported, which was generally attributed to the competition for binding sites. A typical example is wheat ash reported by Wang and co-workers^51^, which was used to investigate effects of heavy metal on the sorption of 2,4,6-trichlorophenol (TCP), and an obvious suppression of the adsorption of 2,4,6-trichlorophenol was observed by copper and lead, due primarily to competition for adsorption sites [the complexation of Cu(II) and Pb(II) was likely via carboxylic, hydroxylic and phenolic groups of ash, and these same functional groups also reacted with TCP during sorption]. Similar results involving Cu(II) and p-Nitrophenol were also obtained due primarily to competition for adsorption sites^52^. In view of the foregoing, site competition possibly played a primary role in the co-adsorption and preloading adsorption.

### Proposed mechanisms of direct competition on P-MMC

As mentioned above, a possible reaction mechanism for Cu(II) and atrazine adsorption onto P-MMC is proposed in Fig. 6. In single system, Cu(II) may be removed in two ways: complexation with carboxyl and hydroxyl groups of P-MMC as well as direct adsorption onto the pores of ordered mesoporous carbon and iron nanoparticles. And atrazine is also removed by adsorption on the surface of ordered mesoporous carbons as well as the hydrophilic sites of oxygen containing groups (-OH and -COOH) (Hydrogen bond). Therefore, site recognition and competition could occur for the simultaneous removal of atrazine and copper using P-MMC. As seen in binary system, a portion of atrazine on carboxyl and hydroxyl groups could be displaced by Cu(II) in the sequential experiment, because Cu(II) exerts stronger binding affinity than atrazine^40^,^53^. In addition, there existed neither electrostatic repulsion nor electrostatic interaction between atrazine and P-MMC at pH 5.0, indicating that the suppression of Cu(II) for atrazine sorption might be attributed to the direct competition of both adsorbates for similar sites such as hydroxyl and hydroxyl group, not the change of surface charges. Thus, the adsorption of atrazine was suppressed in the binary systems due to the occupation of hydrophilic sites by Cu(II).

In addition, it was initially expected that chemi-complexation of Cu(II) would neutralize the P-MMC surface charge, enhancing the hydrophobic effect, thus increasing the sorption of hydrophobic atrazine. However, in the preloading system, the sorption of atrazine was actually decreased in the presence of Cu(II). This was probably because chem-complexation did not decrease the competitive sorption from water molecules a bit^53^. Two mechanism may therefore be responsible for the diminishing sorption of atrazine in the presence of Cu(II). On the one hand, inner-sphere and outer-sphere complexes are formed through carboxyl and hydroxyl groups and hydration, which occupy part of the surface of P-MMC. On the other hand, the complexed Cu(II) are likely to host one or more hydration shells of dense water^40^,^54^, and these large metal cation hydration shells may intrude or shield the P-MMC hydrophobic and hydrophilic sites (carboxyl and hydroxyl groups), and thus compete with atrazine for surface sites indirectly, resulting in the suppression of atrazine adsorption around the Cu(II)-complexed moieties.

### Influence of ionic strength and humic acid

**Figure 7:**
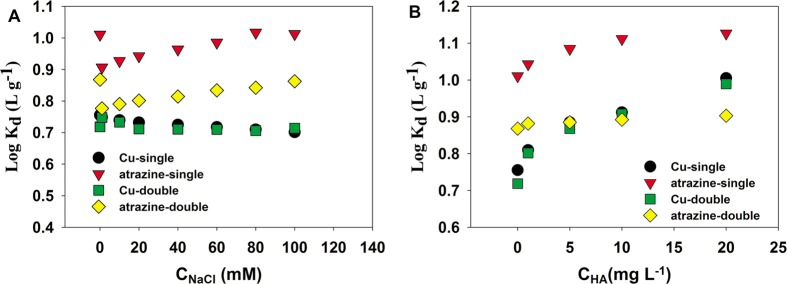
Effects of ionic strength (**A**) and humic acid (**B**) on sorption distribution coefficient (Log *K*_d_) for duplicate points sorption of atrazine and Cu(II) on P-MMC. (initial concentrations of 15 mg·L^^−1^^ and 30 mg·L^−1^ for atrazine and Cu(II), initial pH of 5.0, temperature at 303 K).

The presence of salts and humic acid in atrazine and Cu(II) wastewater may affect the atrazine and Cu(II) sorption. Therefore, the influence of salt ionic and humic acid strength on the removal of atrazine and Cu(II) by P-MMC was studied. As seen in Fig. 7A, the adsorption capacities for Cu(II) slightly decreased with the increase of NaCl concentration in sole and binary systems, which can be well explained that the Cu(II) would shape electrical double-layer complexes with P-MMC, and thus the adsorption would be hindered as the strong-electrolyte NaCl concentration increased. Similar results involving Pb(II) and mesoporous carbon material was reported in our previous work^31^. Moreover, the chemical surface complexation between Cu(II) and carboxyl, hydroxyl groups occurred and also conduced to the removal of Cu(II). Therefore, the small negative effects of ionic strength on Cu(II) sorption were not significant.

However, it is different for atrazine adsorption. Results depicted in Fig. 7A showed an increase in NaCl concentration decreased atrazine uptake firstly, and then increased slightly in sole and binary systems. The possible reason was that the water cluster was formed on the surface of P-MMC by hydrogen bonding with H_2_O molecule with lower NaCl concentration, which could interface and decreased the hydrophobic atrazine uptake. The water cluster would be destroyed at higher NaCl concentration, and it consequently increased atrazine adsorption slightly. Thus, a smaller negative effect occurred on the adsorption amount of atrazine with the lower NaCl concentration.

Relatively strong sorption of humic acid on P-MMC was reported, and the effect of humic acid on the Cu(II) and atrazine sorption on P-MMC was displayed in Fig. 7B. Changing the humic acid concentration from 1 to 20 mg L^−1^, sorption of Cu(II) increased slightly in sole and binary systems. The small positive effect on the adsorption amount of Cu(II) could be explained by the hydrophilic fractions of humic acid with various functional groups, such as carboxyl, phenol, hydroxyl, amine and quinine groups which can bind heavy metals^55^,^56^,^57^,^58^.

Similar phenomenon was found for atrazine uptake. Changing the humic acid concentration from 1 to 20 mg L^−1^, sorption of atrazine increased slightly. The positive effect caused by humic acid on atrazine may be explained by that the common functional groups such as carboxyl, phenol, hydroxyl, amine and quinine groups from humic acid have strong bond ability with atrazine by hydrogen bond. Similar results involving Cu(II) and p-Nitrophenol were also obtained due primarily to competition for adsorption sites^10^.

### Regeneration and leaching experiments

The regeneration of P-MMC was conducted by adding Cu(II)/atrazine-loaded P-MMC solids to 10 mL 20% acidic ethanol solution, and the mixture was stirred at 25 °C and 150 rpm for 24 h. The results were showed in Fig. S-6, Supporting Information, after five sorption/desorption cycles, the adsorption efficiency remained at high level above 81% for Cu(II) and 89% for atrazine, indicating the relatively excellent adsorption stability of P-MMC. Hence, in consideration of its regeneration abilities and magnetic separation, P-MMC improved the potential for the removal of organics and heavy metals in more complex environments by *in-situ* regeneration and reuse. In addition, as shown in Fig. S-7, the leaching of Fe from P-MMC was slightly less than MMC, and the leaching of Fe from P-MMC in regeneration process was not serious (Fig. S-8), which may be ascribed to the surface modification of polyacrylic acid.

### Application in real water samples

**Figure 8:**
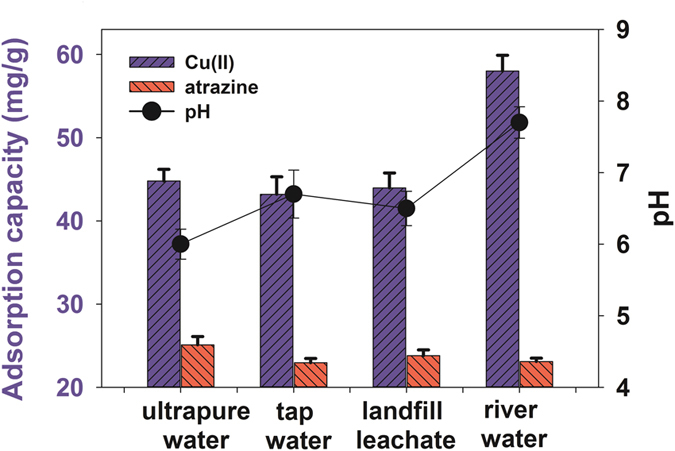
The practical application of P-MMC to real samples (initial concentrations of 15 mg·L^−1^ and 30 mg·L^−1^ for atrazine and Cu(II), initial pH of 5.0, temperature at 303 K).

P-MMC was applied to treat real environmental samples including tap water, river water and landfill leachate to investigate its practical application, and the results were shown in Fig. 8. It was observed that the sorption capacity for Cu(II) in tap water and landfill leachate were less than that in lab ultrapure water. It may be that there existed many common cations [e.g., Na^+^, Ca^2+^, Mg^2+^, and K^+^] in these water samples. These cations not only occupied the adsorption sites of P-MMC, but also impacted ionic strength, aggrandizing the contact difficulty between Cu(II) and P-MMC. In addition, the Cu(II) uptake amount in landfill leachate was a little higher than tap water, due to plentiful organic matters in landfill leachate, such as humic acid and fulvic acid, and thus improved Cu(II) adsorption slightly. Moreover, the Cu(II) uptake amount in river water was the highest, due to higher pH was favorable to Cu(II) adsorption by precipitation and electrostatic attraction^26^,^59^,^60^.

As for atrazine, it was observed that the sorption capacity in tap water, river water and landfill leachate were less than that in lab ultrapure water. It might be attributed to the coexisting cations [e.g., Pb^2+^ Mg^2+^, and Cd^2+^] in real water samples, which may compete with atrazine for the same adsorption sites. The atrazine uptake amount in landfill leachate was a little higher than tap water and river water, due to plentiful organic matters in landfill leachate, such as humic acid and fulvic acid, and thus improved atrazine adsorption slightly. The sorption capacities for atrazine and Cu(II) in real samples were all more than 91.47% and 96.43% of those in lab ultrapure water, respectively (Fig. 8). These results indicated that P-MMC exhibited relatively superior behavior in removing atrazine and Cu(II) in real samples.

## Conclusions

Ordered mesoporous carbon combining properties of magnetism and polyacrylic acid were synthesized for the simultaneous removal of Cu(II) and atrazine. P-MMC contained both hydrophilic and hydrophobic sites and exhibited relative excellent adsorption of Cu(II) and atrazine. The results showed that site recognition and site competition were the predominant adsorption mechanisms. Humic acid and ionic strength exerted some positive influence on the removal of atrazine and Cu(II). Besides, the regeneration studies revealed that P-MMC could be effectively recovered with acidic ethanol. The superior properties demonstrated by P-MMC indicated that it could be applied to wastewaters containing both Cu(II) and atrazine.

## Methods

### Preparation of P-MMC and characterization of materials

The mesostructured SBA-15 silica template was synthesized as described previously in our laboratory^31^,^61^,^62^. Magnetic ordered mesoporous nanocomposite (MMC) was synthesized by following a co-impregnation method with slight alterations^63^. Details about the preparation of MMC can be seen in Text S-1, Supporting Information (SI). Besides, P-MMC was synthesized as described previously in our laboratory^37^. The structural information for P-MMC, including transmission electron microscopy (TEM) images are shown in Fig. S-1, and the detail description can be seen in Supporting Information. Furthermore, our previous work showed that the P-MMC was enriched in carboxyl and hydroxyl group^37^. Thus, these groups and numerous pores on the outside of the OMC implying that P-MMC could contain both hydrophilic and hydrophobic sites^31^,^37^.

### Batch adsorption studies

The equilibrium experiments were performed in a 50 mL stoppered conical flasks in a water bath shaker by mixing 5 mg of adsorbent and 10 mL of solution containing various concentrations of atrazine and Cu(II). The concentration range of atrazine and Cu(II) was 1–30 mg·L^−1^ and 10–150 mg·L^−1^, respectively. The pH of the solution was adjusted to 5.0 with HCl or NaOH, which was determined in the preliminary experiments (Fig. S-2 and Fig. S-3). The flasks were completely sealed and agitated in an incubator shaker at 303 K and 150 rpm for 24 h. Then, the concentrations of atrazine and Cu(II) in the residual aqueous phase were determined by HPLC (Agilent 1100, USA) equipped with an UV-vis photodiode array detector and a Perkin-Elmer Analyst 700 atomic absorption spectrophotometer (AAS, Perkin-Elmer, USA), respectively. Experiments were carried out in duplicate, and the arithmetic mean values were calculated, with the standard deviations less than 5%.

In the preloading adsorption experiments, fresh P-MMC was first equilibrated with 1–30 mg·L^−1^ of atrazine or 10–150 mg·L^−1^ of Cu(II), and then the preloaded P-MMC was conversely fed by Cu(II) or atrazine at various concentrations to obtain a second quilibrium.

### Stability and regeneration studies

To carry out the leaching experiment, 5 mg MMC or P-MMC was dispersed in 10 mL of Cu(II) solutions with different pH. At the specific contact time, the suspension was separated and the leached iron concentration in the supernatant was determined by a flame atomic absorption spectrometer (FAAS, PerkinElmer AA700, USA). The feasibility of regenerating P-MMC for repeated use was investigated by using acidic ethanol. Specifically, after accomplishment of the adsorption experiments, the Cu(II)-loaded (or atrazine-loaded) adsorbent was magnetically separated and subsequently added into 10 mL 20% acidic ethanol for desorption at 150 rpm at 303 K. After washing thoroughly with ultrapure water to neutral pH, the regenerated adsorbent was recycled and reused, and the adsorption performance of P-MMC was investigated again, meanwhile, leached iron concentration in the supernatant was also determined.

### Application of P-MMC to real water samples

Tap water (from Changsha No. 2 Drinking Water Company), river water (from Xiangjiang, Hunan), and landfill leachate (from a municipal solid waste landfill in Changsha, China) samples were used as real environmental sample in this study to test the efficiency of P-MMC. Specifically, 5 mg P-MMC was added into different initial concentrations of Cu(II) or atrazine solution for adsorption test at 303 K and 150 rpm for 24 h. Then, the concentrations of atrazine and Cu(II) in the residual aqueous phase were determined.

### Goodness-of-fit measure (GoFM)

The model parameters for Freundlich and Langmuir isotherm have been determined by minimizing the difference between the experimental and modeled *Q*_e_ values (through the sum of the absolute errors (EABS) and the average relative error (ARE)) using the iterative method^64^.

The sum of the absolute errors (EABS) model:


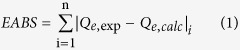


The average relative error (ARE)


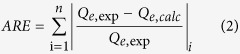


“exp” and “calc” show the experimental and calculated values.

## Additional Information

**How to cite this article:** Zhou, Y. *et al*. Simultaneous removal of atrazine and copper using polyacrylic acid-functionalized magnetic ordered mesoporous carbon from water: adsorption mechanism. *Sci. Rep.* **7**, 43831; doi: 10.1038/srep43831 (2017).

**Publisher's note:** Springer Nature remains neutral with regard to jurisdictional claims in published maps and institutional affiliations.

## Supplementary Material

Supporting Information
