# Supplementary material for: Simultaneous removal of atrazine and copper using polyacrylic acid-functionalized magnetic ordered mesoporous carbon from water: adsorption mechanism
Source: Sci Rep. 2017 Mar 2;7:43831. doi: 10.1038/srep43831 (PMC5333147; doi:10.1038/srep43831)
Supplement: Supporting Information [file srep43831-s1.doc]

**Supporting Information (SI)**

**Simultaneous removal of atrazine and copper using polyacrylic acid-functionalized magnetic ordered mesoporous carbon from water: adsorption mechanism**

Yaoyu Zhou^a,b^, Fengfeng Zhang^a^, Lin Tangb,c,[[1]](#footnote-2)*, Jiachao Zhang^a^, Guangming Zengb,c,[[2]](#footnote-3)*, Lin Luo^a^, Yuanyuan Liu^d^, Pei Wang^b,c^, Bo Peng^b,c^, Xiaocheng Liu^a^

^a^ College of Resources and Environment, Hunan Agricultural University, Changsha 410128, China;

^b^ College of Environmental Science and Engineering, Hunan University, Changsha 410082, China;

^c^ Key Laboratory of Environmental Biology and Pollution Control, Ministry of Education, Hunan University, Changsha 410082, China;

^d^ Department of Resources and Environmental Sciences, Changsha University of Science and Technology, Changsha 410114, China.

The SI contains 24 total pages with 8 Figures and 5 Tables

**List of Supplemental Figures**

**Fig. S-1**. TEM images of OMC (A), MMC (B), P-MMC (C).

**Fig. S-2**. Effects of pH on sorption distribution coefficient (Log *K*_d_) for duplicate points sorption of atrazine or/ and Cu(II) on P-MMC (initial concentrations of 15 mg·L-1 and 30 mg·L-1 for atrazine and Cu(II), initial pH from 2.0 to 6.0, temperature at 303 K).

**Fig. S-3**. Zeta potential curves of P-MMC and MMC as a function of solution pH.

**Fig. S-4.** Mutual effect of atrazine and Cu(II) adsorption onto P-MMC; (A) Langmuir isotherms for atrazine with interaction to Cu(II) of different initial concentrations tested at 303 K, with initial pH of 5.0; (B) Freundlich isotherms for Cu(II) with interaction to atrazine of different initial concentrations tested at 303 K, with initial pH of 5.0.

**Fig. S-5.** Plot of ln *K*_d_ vs 1/T obtained for the adsorption of (A) atrazine and (B) Cu(II) onto P-MMC.

**Fig. S-6.** Five consecutive adsorption–desorption cycles of P-MMC for Cu(II) and atrazine. Initial Cu(II) and atrazine concentrations of 30 mg·L^-1^ and 15 mg·L^-1^, respectively, adsorbent dose of 5 mg, pH 5.0, temperature of 303 K, contact time of 24 h.

**Fig. S-7.** Effect of initial pH on the concentrations of dissolved metal ions. MMC or P-MMC dosage of 5 mg, the concentration of Cu(II) as 30 mg·L^-1^, temperature of 303 K, contact time of 24 h.

**Fig. S-8** The concentrations of dissolved metal ions in the adsorption–desorption cycles. P-MMC dosage of 5 mg, the concentration of Cu(II) as 30 mg·L^-1^, pH 5.0, temperature of 303 K, contact time of 24 h.

**List of Supplemental Tables**

**Table S-1** Adsorption isotherm parameters for adsorption of atrazine and Cu(II) onto different OMC, and the main characteristics of different OMC.

**Table S-2** The isotherm models of various adsorbents for the removal of atrazine.

**Table S-3** Comparison of adsorption capacities for adsorption of Cu(II) and/or atrazine by various adsorbents reported in the literature.

**Table S-4** Adsorption isotherm parameters for adsorption of atrazine and Cu(II) onto P-MMC.

**Table S-5** Thermodynamic parameters for atrazine and Cu(II) adsorption on P-MMC.

**Text S-1:**

**Preparation of** **mesoporous carbon-based materials**

Magnetic mesoporous carbon synthesized under optimized conditions based on the method mentioned before . Typically, multi-component solution containing Fe(NO_3_)_3_·9H_2_O, 0.5 mL ethanol, 0.8 mmol Ni(NO_3_)_2_·6H_2_O and 1 mL furfuryl alcohol was prepared. After infiltrating 1.4 mL of the solution into 1.2 g SBA-15 template, the impregnated mixture was cured at 80 ^o^C in air for 10 h, followed by carbonization and reduction of the metal oxides under a 5% H_2_-95% Ar atmosphere at 900 ^o^C for 2 h. The resultant product was washed by heated 2 M NaOH solution and water, respectively, and dried at 70 ^o^C to get magnetic mesoporous carbon (MMC). Besides, for comparison, pristine ordered mesoporous carbon (OMC) was also prepared without iron and nickel. Besides, P-MMC was synthesized under optimized conditions based on the method mentioned before [^3^](#_ENREF_3). Typically, 0.5 g of MMC was mixed with polyacrylic acid (PAA) under pH 5.0 and agitated for 24 h. The mixture was then ﬁltered, washed and then stored for the future use.

**Text S-2: The isotherm models**

All measurements were performed in triplicate, and average values and standard deviations were presented. The amount of adsorbed atrazine or Cu(II) was calculated by Eq. (1):

$Q_{e}=\frac{\left(C_{0}-C_{e}\right)\times V}{m}$ (1)

where *Q_e_* is the equilibrium adsorption capacity (mg/g); *C_0_* and *C_e_* are the initial and equilibrium concentration of atrazine or Cu(II) in solution (mg/L), respectively; *V* is the volume of aqueous solution (mL) and *m* is the mass of adsorbent used (mg).

**Langmuir model**

Langmuir model was applied to described a monomolecular adsorption without interaction between the adsorbed molecules with a finite number of active centers with the same energy [^4^](#_ENREF_4). The constant parameters of the Langmuir equation have a strictly defined physical meaning in contrast to the parameters of the empirical Freundlich equation . The relevant isotherm equation is represented as Eq. (2):

$Q_{e}=\frac{Q_{m}K_{L}C_{e}}{1+K_{L}C_{e}}$ (2)

The linearized form of the Langmuir equation is:

$\frac{C_{\text{e}}}{Q_{e}}=\frac{1}{Q_{m}K_{L}}+\frac{C_{e}}{Q_{m}}$ (3)

where *Q_m_* (mg/g) is the maximum amount of adsorption corresponding to the monolayer coverage, and *K_L_* (L/mg) is the Langmuir constant related to the adsorption energy.

**Freundlich** **model**

The Freundlich model was originally used without theoretical justification, later it was

found that it represents the relationship between a non-ideal and reversible adsorption [^7^](#_ENREF_7). It is applied to heterogeneous surfaces with interaction between the adsorbed molecules (multilayer) and assumes an exponential energy distribution of the active adsorption sites [^8^](#_ENREF_8).

The Freundlich adsorption equation is given as Eq.4:

$Q_{\text{e}}=K_{F}C_{\text{e}}^{1/n}$ (4)

The linearized form of the Freundlich equation is:

$L\text{og}Q_{\text{e}}=L\text{og}K_{F}+\frac{1}{\text{n}}L\text{og}C_{\text{e}}$ (5)

where, *K*_F_ is the adsorption capacity in the unit concentration,1/n is the intensity of adsorption, *C*_e_ is the equilibrium concentration in mg/L, and *Q*_e_ is the amount of atrazine and Cu(II) adsorbed at equilibrium in mg/g. *K*_F_ and 1/n can be specified by the linear diagram of Log*Q*_e_ against Log*C*_e_. 1/n represents the type of isotherm, where if 1/n < 0, then it is irreversible, 0 <1/n < 1, it is desirable, and if 1/n > 1, it is undesirable [^9^](#_ENREF_9).

**The distribution coefficients**

The sorption distribution coefficient (*K*_d_, L g^-1^) is defined by the ratio of atrazine or Cu(II) adsorbed per unit sorbent mass (*Q*_e_, mg g^-1^) and the equilibrium adsorbate concentration (*C*_e_, mg L^-1^). The distribution coefficients are mass weighted partition coefficients of targeted pollutant between the solid and solution phases and are calculated by Equation 6 [^10^](#_ENREF_10):

$K_{\text{d}}=\frac{Q_{\text{e}}}{C_{\text{e}}}=K_{F}C_{\text{e}}^{_{\frac{1}{n\text{-}1}}}$ (6)

**Thermodynamic of adsorption**

Thermodynamics were utilized to evaluate the adsorption behaviors of N-MMC, and the corresponding equations are expressed as Eq. (9)-(10):

$\text{ln}(\frac{Q_{e}}{C_{e}})=\frac{\Delta S^{0}}{R}-\frac{\Delta H^{0}}{RT}$ (7)

$\Delta G^{0}=\Delta H^{0}-T\Delta S^{0}$ (8)

where *R* is the gas constant (8.314 J/mol/K), *T* is the absolute temperature (K), and *ΔS* (J/mol/K), *ΔH* (kJ/mol) and *ΔG* (kJ/mol) are the changes in the entropy, enthalpy and Gibb’s free energy of the system, respectively.

**Characterization of mesoporous carbon-based materials**

The characterization data of Transmission electron microscopy (TEM, JEOL JEM-1230), Fourier transform infrared spectrometer (FTIR spectrometer Nicolet NEXUS 670) and nitrogen adsorption measurements (ASAP 2020 volumetric adsorption analyzer) were shown in Fig. S-1 and our previous work ^2^. TEM image showed that OMC had well-ordered mesopore arrays and 2D hexagonal pore structure. From the TEM image of Fig. S-1B, large domains of ordered stripe-like structures were observed for MMC, and magnetic nanoparticles were found embedded in the carbon rods over the entire MMC particles, and the nanoparticles with an average diameter about 10 nm were dispersed on the carbon matrix. Large voids resulted from some broken nanorods (indicated with arrows) could also be observed, corresponding to the secondary mesopores of the composite [^11^](#_ENREF_11). As demonstrated in Fig. S-1C, P-MMC has retained the 2-D hexagonal mesostructured of MMC, and nanoparticles and voids were also found in P-MMC, indicating the PAA chelating did not destroy the structure of MMC. Furthermore, the N_2_ sorption isotherms with the corresponding pore distribution curves were described in our previous work ^2^, which indicated the corresponding pore size distributions, showing two pore systems centered at 4.0 nm and 18.0 nm, the other at 3.0 nm and 5.0 nm for MMC and P-MMC, respectively. This result also confirmed the existence of bimodal systems. The BET surface areas results indicated that polyacrylic acid was successfully modified inside the pores of MMC ^2^. In addition, FTIR of P-MMC was described in our previous work ^2^, and the results demonstrated that the P-MMC was enriched with oxygen-containing functional groups (carboxyl and hydroxyl groups)

**Influence of pH**

The effect of pH on the adsorption of atrazine and Cu(II) was illustrated in Fig. S-2. It was found that the Log*K*_d_ of atrazine and Cu(II) by P-MMC was the lowest at pH 2.0, pH 3.0 and pH 4.0, but remained almost constant above pH 5.0 (5.0–6.0). At pH 2.0-pH 4.0 where atrazine is protonized (Atrazine is a weak base, with a p*K*_a_ of 1.7. When pH is near its p*K*_a_, half of atrazine is present in the cationic form while the other half stays in the non-ionic form), Cu(II) is positively charged, and P-MMC is also positively charged (see Fig. S-3), the electrostatic repulsion between atrazine/Cu(II) and P-MMC makes atrazine/Cu(II) adsorption unfavorable. However, at pH 4.0-pH 6.0, atrazine exists almost exclusively as neutral molecules, Therefore, there existed neither electrostatic repulsion nor electrostatic interaction between atrazine and P-MMC at pH 5.0. And for Cu(II), over the pH 6.0, The precipitation of Cu(II) ions may contribute to high uptake of the metal ions. Hence, high pH values were not studied. And thus other experiments to perform atrazine and Cu(II) removal by the material were conducted at pH 5.0.

**Thermodynamic investigations**

The thermodynamic investigations were performed in a 50 mL stoppered conical flasks in a water bath shaker by mixing 5 mg of adsorbent and 10 mL of solution containing atrazine and Cu(II), and then agitated in an incubator shaker at the 293 K, 303 K and 313 K, respectively. Plot of ln *K*_d_ vs 1/T obtained for the adsorption of atrazine and Cu(II) onto P-MMC are shown in Figure S-5.


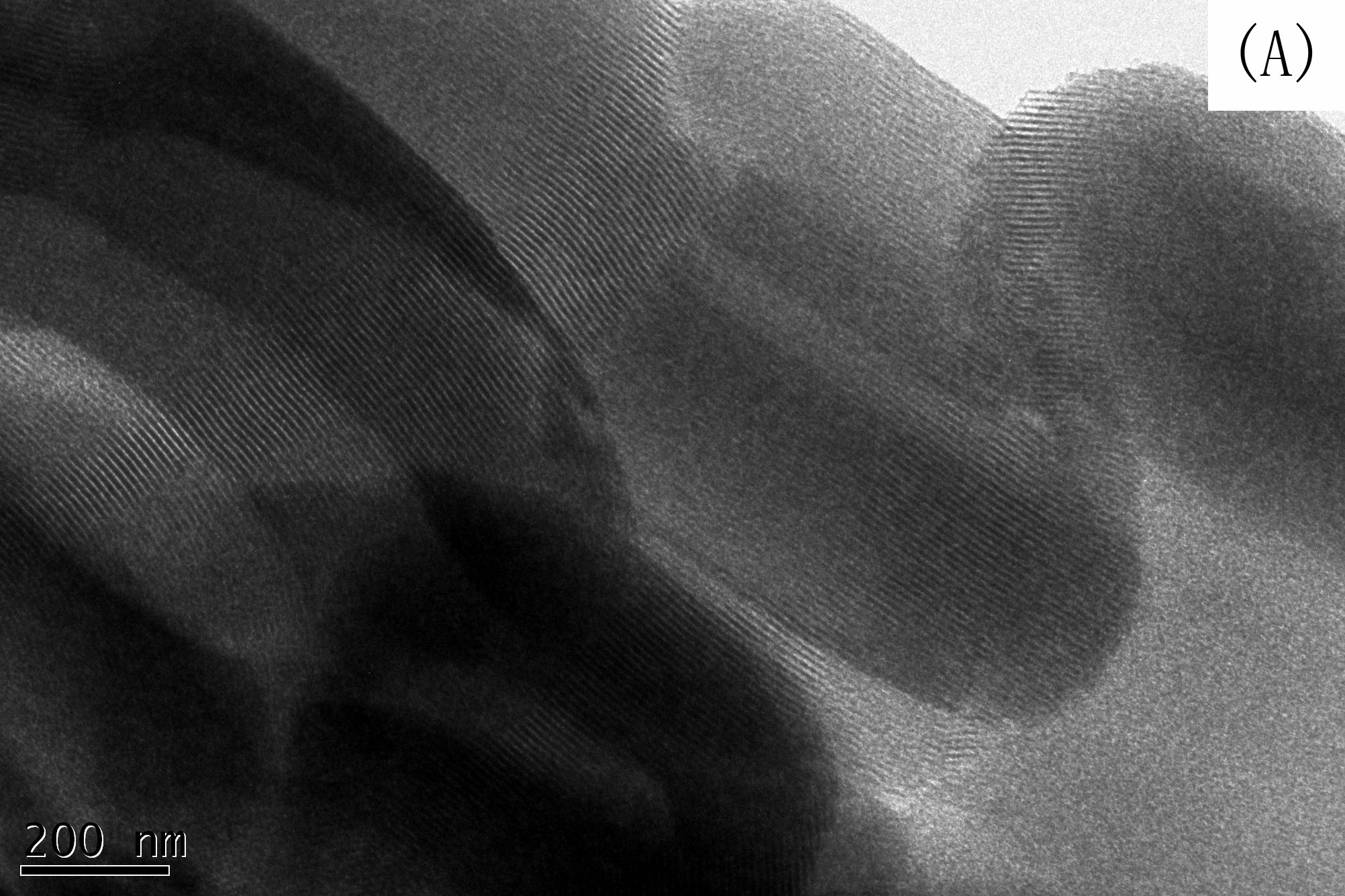

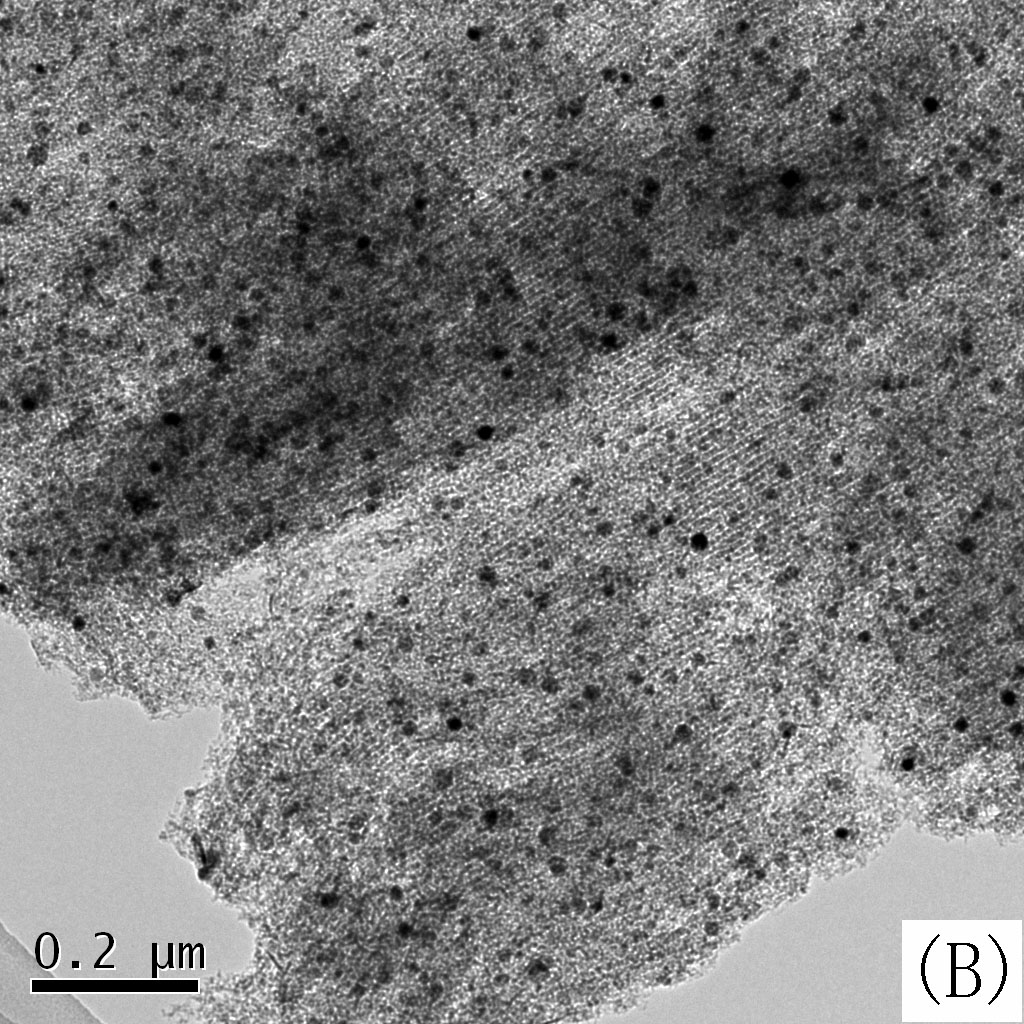


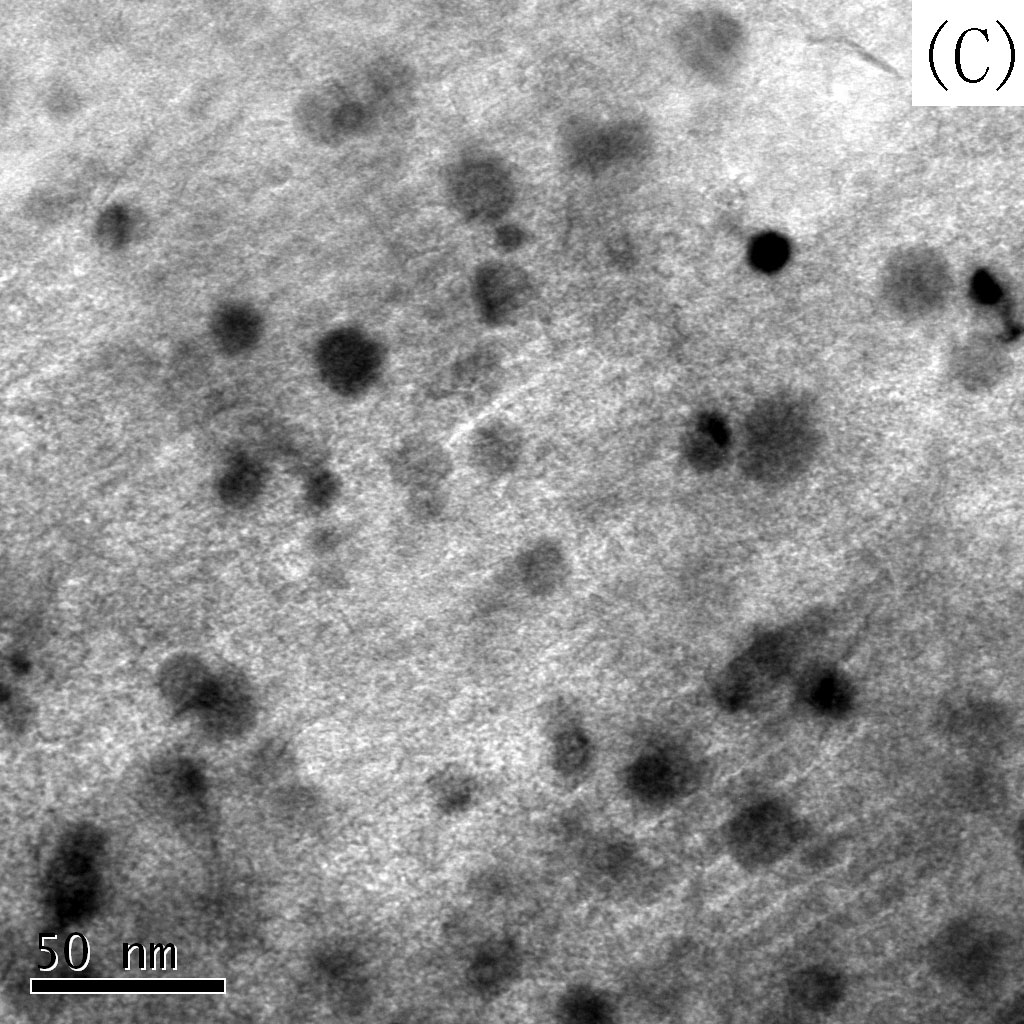


**Fig. S-1**

**Fig. S-2**


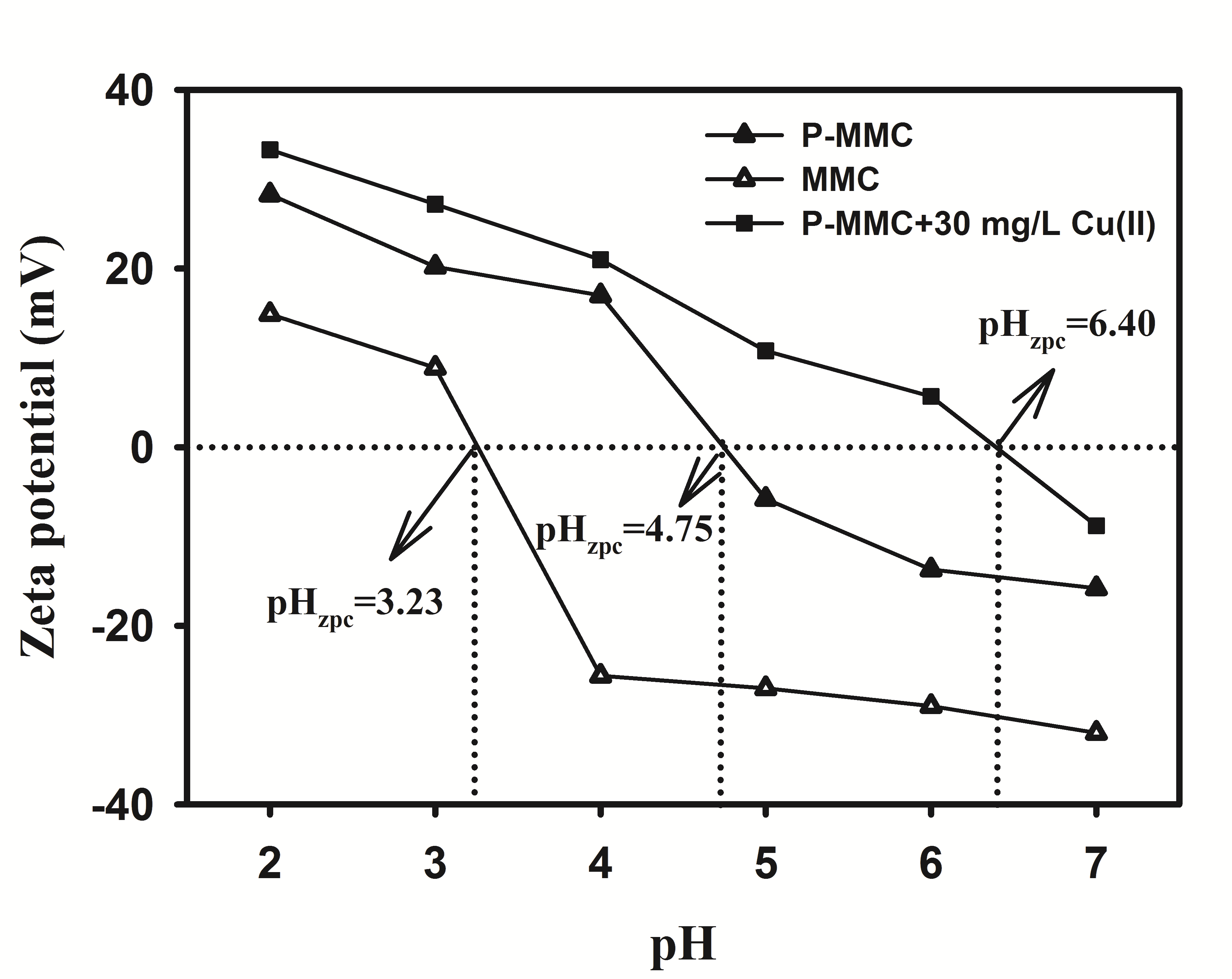


**Fig. S-3**


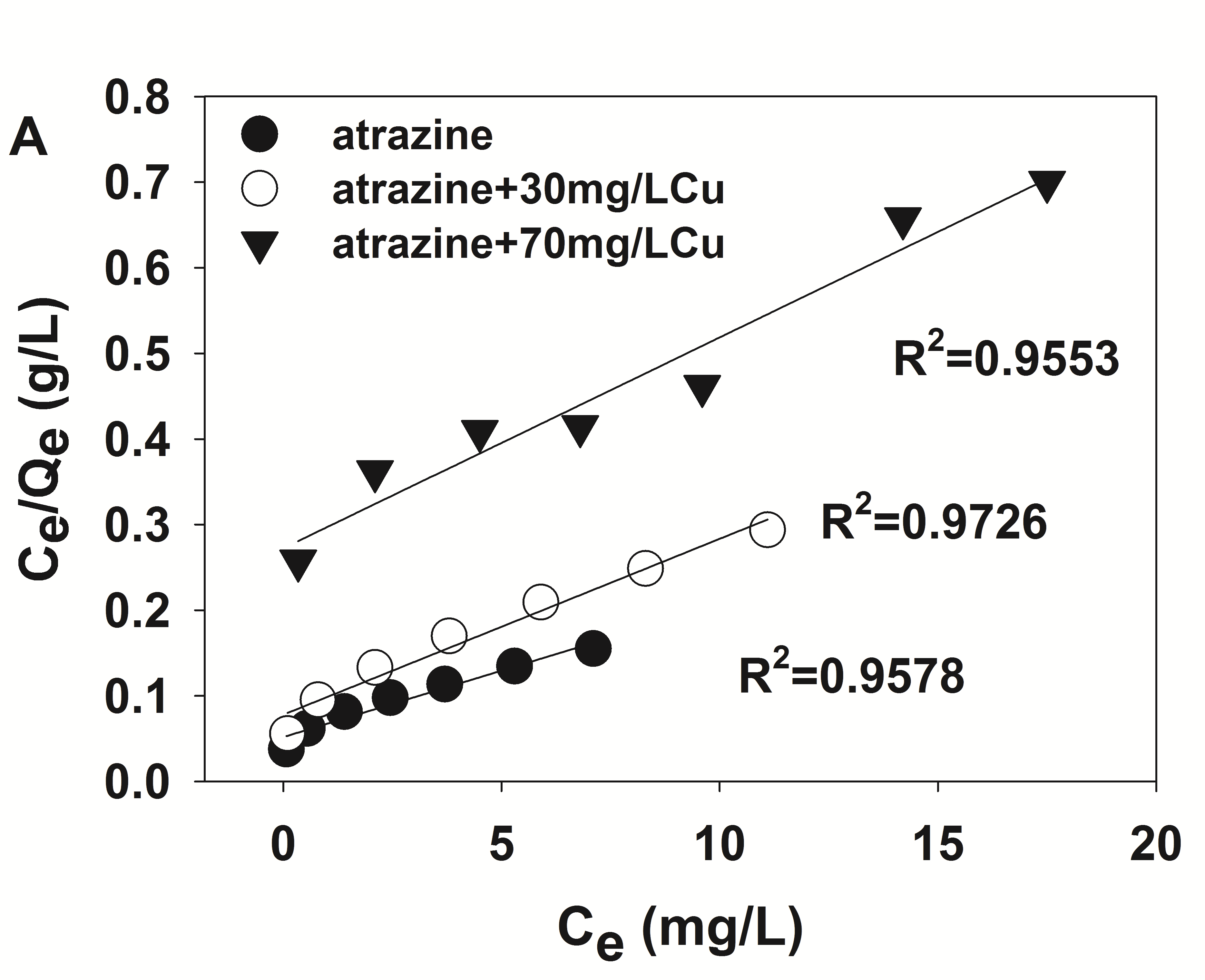


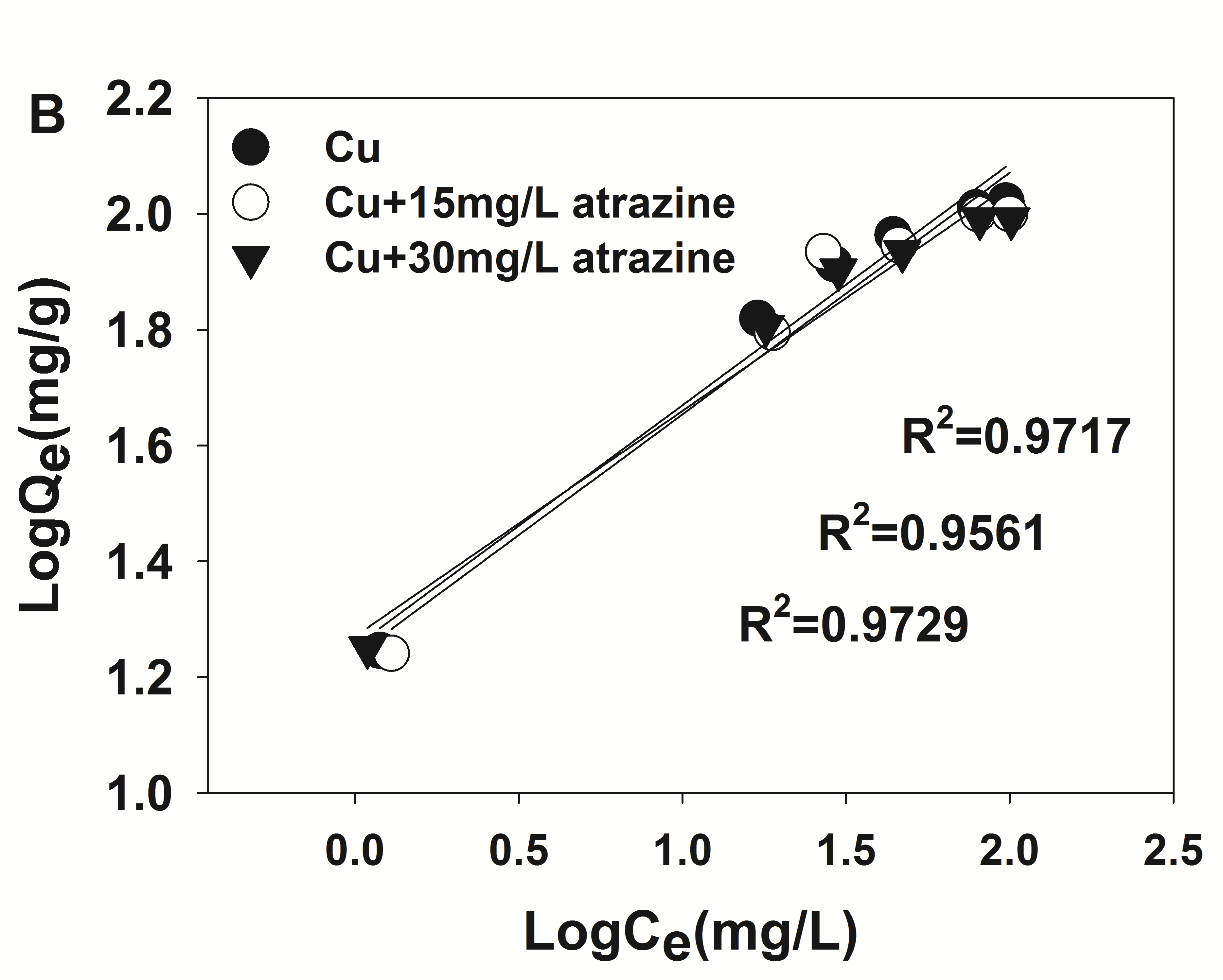


**Fig. S-4**

**Fig. S-5**


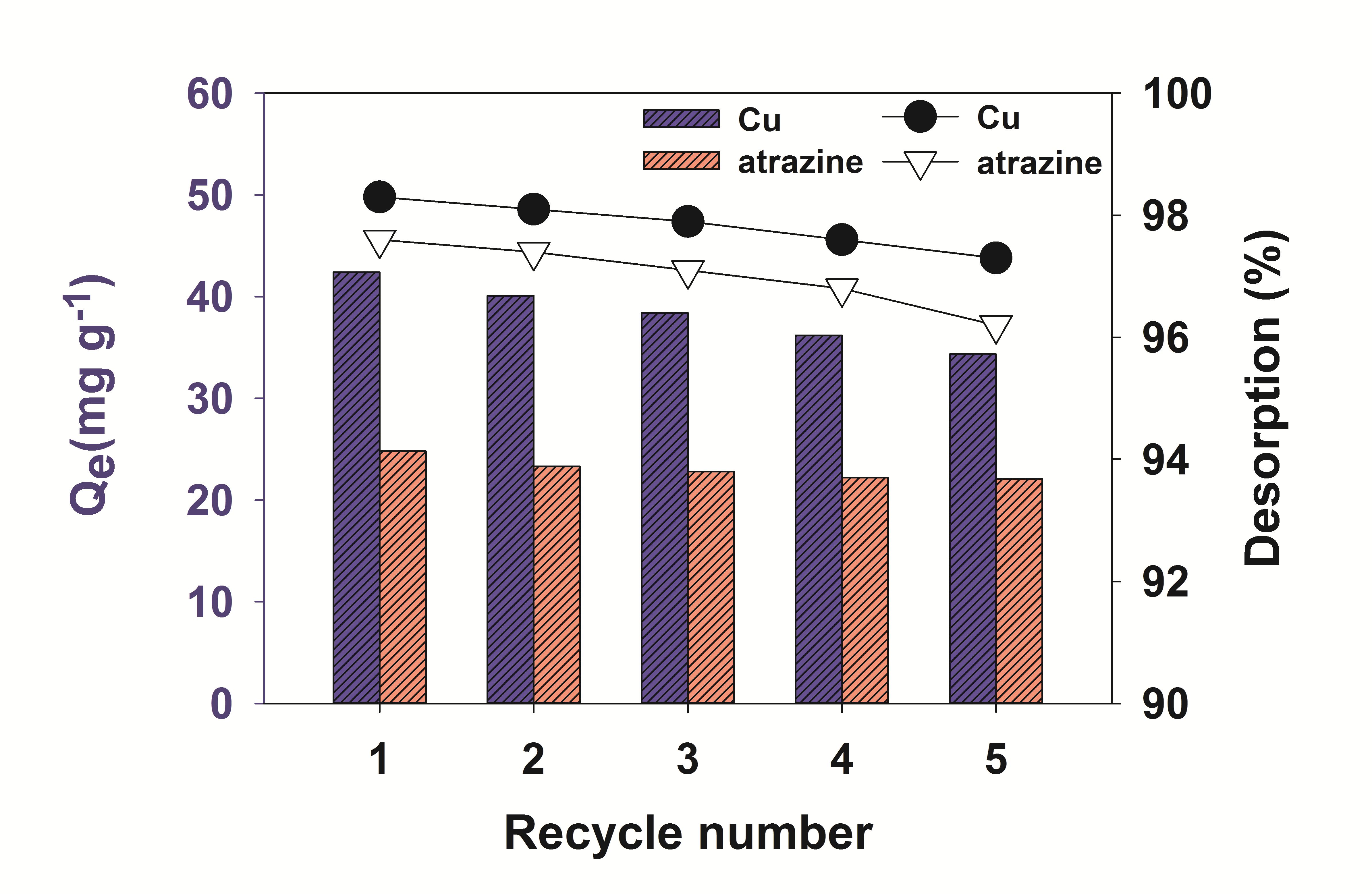


**Fig. S-6**


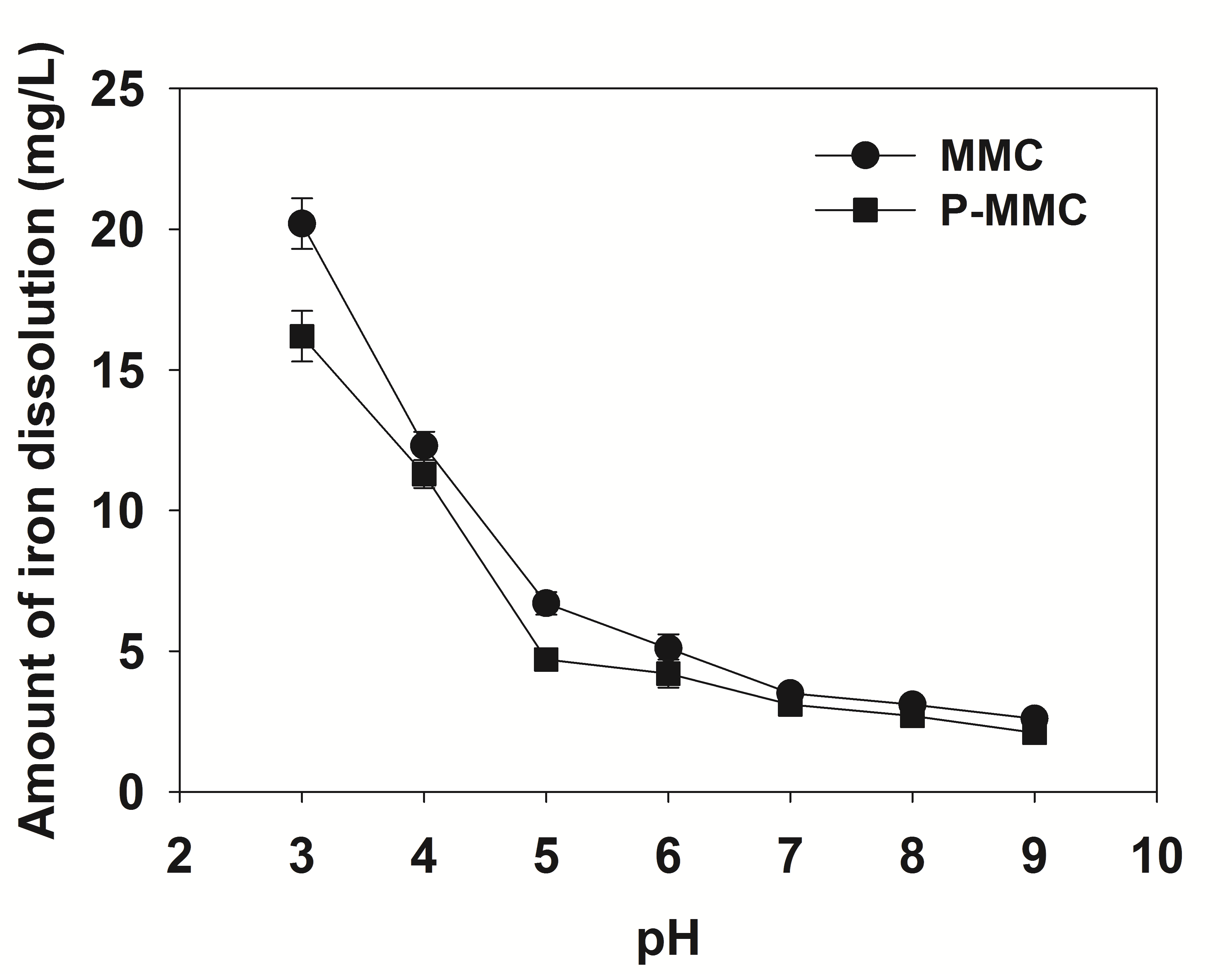


**Fig. S-7**


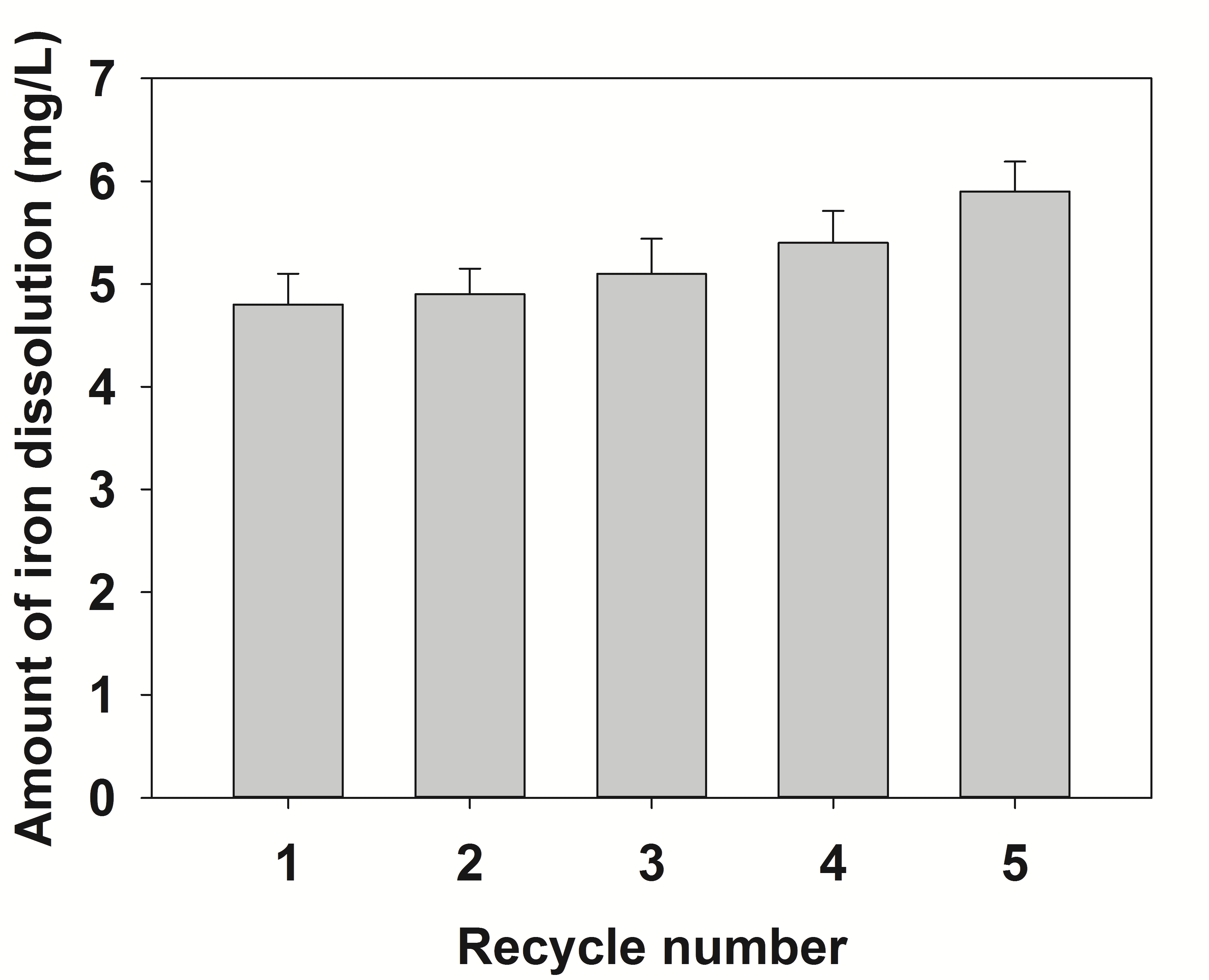


**Fig. S-8**

**Table S-1 Adsorption isotherm parameters for adsorption of atrazine and Cu(II) onto different OMC, and the main characteristics of different OMC ^[2]^**.

| Materials | Pore size (nm) | Specific Surface area (m^2^/g) | The main Functional groups | Isoelectric point | Absorbates | Langmuir | | | Freundlich | | |
| --- | --- | --- | --- | --- | --- | --- | --- | --- | --- | --- | --- |
|  |  |  |  |  |  | *K_L_*  (L/mg) | *Q_max_*  (mg/g) | *R^2^* | K_f_ (L/mg) | 1/n | *R*^2^ |
| OMC | 4.75 | 1231.5 | -OH | 4.8 | atrazine | 0.1130 | 49.74 | 0.9764 | 6.6470 | 0.5881 | 0.9943 |
|  |  |  |  |  | Cu(II) | 0.0334 | 79.93 | 0.9921 | 9.0229 | 0.4219 | 0.9417 |
| MMC | 5. 18 | 1058.7 | -OH | 3.23 | atrazine | 0.1089 | 62.19 | 0.9292 | 7.7859 | 0.6187 | 0.9993 |
|  |  |  |  |  | Cu(II) | 0.0353 | 95.21 | 0.9916 | 11.0022 | 0.4218 | 0.9565 |
| P-MMC | 3.5 | 689.7 | -OH | 4.75 | atrazine | 0.2058 | 76.51 | 0.9578 | 14.1879 | 0.6086 | 0.9967 |
|  |  |  | -COOH |  | Cu(II) | 0.0809 | 118.13 | 0.9977 | 25.0105 | 0.3294 | 0.9687 |

**Table S-2 The isotherm models of various adsorbents for the removal of atrazine.**

| Adsorbent | Freundlich model  *R^2^* | Langmuirmodel  *R^2^* | References |
| --- | --- | --- | --- |
| Activated carbons | 0.948 | 0.974 | [12] |
| Nyex 100 | 0.980 | - | [13] |
| Humic acid–silica gel mixtures | 0.996 | - | [14] |
| sewage sludge amended luvisol soil | 0.977 | - | [15] |
| humic acids coated nanoparticles | 0.968 | - | [16] |
| magnetic multi-walled carbon nanotube | 0.990 | 0.980 | [17] |
| Sludge derived biochars | 0.970 | 0.900 | [18] |
| Surfactant-modified bentonite clays: | 0.996 | - | [19] |
| P-MMC | 0.9967 | 0.9578 | This work |

"-" stands for No fitting.

**Table S-3. Comparison of adsorption capacities for adsorption of Cu(II) and/or atrazine by various adsorbents reported in the literature.**

| Adsorbent | Cu(II) | |  | | atrazine | | | Refs. | |
| --- | --- | --- | --- | --- | --- | --- | --- | --- | --- |
|  | pH | Q_m_(mg/g) | |  | | pH | Q_m_(mg/g) | |  |
| Commercial activated carbon | 5.0 | 14.78 | |  | | - | - | | 20 |
| Activated carbon prepared from hazelnut husks | 5.7 | 6.645 | |  | | - | - | | 21 |
| Activated carbon prepared from Ceiba pentandra hulls | 6.0 | 20.80 | |  | | - | - | | 22 |
| Chitosan crosslinked with epichlorohydrin-triphosphate | 6.0 | 130.72 | |  | | - | - | | 23 |
| Amino functionalized multi-walled carbon nanotubes | 5.5 | 20.1 | |  | | - | - | | 24 |
| Commercial activated carbons | - | - | |  | | 5.0 | 24.51 | | 12 |
| Commercial activated carbons | - | - | |  | | 6.0 | 31.70 | | 25 |
| CS450^a^ | - | - | |  | | 7.0 | 5.52 | | 26 |
| ADPCS450^b^ | - | - | |  | | 7.0 | 53.85 | | 26 |
| Magnetic multi-walled carbon nanotube | 6.0 | 38.91 | |  | | 6.0 | 40.16 | | 17 |
| P-MMC | 5.0 | 117.50 | |  | | 5.0 | 76.51 | | This work |

a, biochars from corn straw; b, biochars modified with ammonium dihydrogen phosphate; "-" stands for No test.

**Table S-4 Adsorption isotherm parameters for adsorption of atrazine and Cu(II) onto P-MMC.**

| Adsorbates | Langmuir | | | Freundlich | | |
| --- | --- | --- | --- | --- | --- | --- |
|  | *K_L_* (L/mg) | *Q_max_* (mg/g) | *R^2^* | *K*_F_(L/mg) | 1/n | R^2^ |
| atrazine | 0.2058 | 76.51 | 0.9578 | 14.1879 | 0.6086 | 0.9967 |
| atrazine+30mg/L Cu | 0.1889 | 54.88 | 0.9726 | 10.4488 | 0.5448 | 0.9933 |
| atrazine+70mg/L Cu | 0.0947 | 39.97 | 0.9553 | 4.7574 | 0.5915 | 0.9863 |
| Cu | 0.0820 | 117.50 | 0.9966 | 25.8970 | 0.3183 | 0.9717 |
| Cu+15mg/L atrazine | 0.0887 | 114.18 | 0.9945 | 26.1384 | 0.3075 | 0.9561 |
| Cu+30mg/L atrazine | 0.0896 | 112.10 | 0.9957 | 25.9183 | 0.3023 | 0.9729 |

**Table S-5** Thermodynamic parameters for atrazine and Cu(II) adsorption on P-MMC.

| Adsorbates | Concentrations | Δ*H^0^*  (kJ/mol) | Δ*S^0^*  (J/K·mol) | Δ*G^0^* (kJ/mol) | | |
| --- | --- | --- | --- | --- | --- | --- |
|  |  |  |  | 293K | 303K | 313K |
| Atrazine  (mg/L) | 10 | -5.27 | 3.51 | -6.30 | -6.34 | -6.37 |
|  | 15 | -4.41 | 4.82 | -5.82 | -5.88 | -5.92 |
|  | 20 | -3.92 | 5.11 | -5.42 | -5.47 | -5.52 |
| Cu(II)  mg/L | 30 | 29.785 | 112.33 | -3.13 | -4.25 | -5.37 |
|  | 60 | 30.87 | 110.25 | -1.43 | -2.53 | -3.64 |
|  | 90 | 30.13 | 106.56 | -1.09 | -2.16 | -3.22 |

**References**

1. Z. Wang, X. Liu, M. Lv and M. Jian, *Carbon*, 2010, **48**, 3182-3189.

2. G. Zeng, Y. Liu, T. Lin, G. Yang, Y. Pang, Z. Yi, Y. Zhou, L. Zhen, M. Li and M. Lai, *Chemical Engineering Journal*, 2015, **259**, 153-160.

3. H. I. Lee, Y. Jung, S. Kim, J. A. Yoon, H. K. Jin, J. S. Hwang, M. H. Yun, J. W. Yeon, S. H. Chang and M. K. Ji, *Carbon*, 2009, **47**, 1043-1049.

4. M. H. Dehghani, M. Ghadermazi, A. Bhatnagar, P. Sadighara, G. Jahed-Khaniki, B. Heibati and G. Mckay, *Journal of Environmental Chemical Engineering*, 2016, **4**, 2647-2655.

5. G. Mckay, A. Mesdaghinia, S. Nasseri, M. Hadi and M. S. Aminabad, *Chemical Engineering Journal*, 2014, **251**, 236-247.

6. P. Hadi, M. H. To, C. W. Hui, C. S. K. Lin and G. Mckay, *Water Research*, 2015, **73C**, 37-55.

7. X. Chen, *Information*, 2015, **6**, 14-22.

8. P. Hadi, J. Guo, J. Barford and G. Mckay, *Environmental Science & Technology*, 2016.

9. B. Heibati, S. Rodriguez-Couto, A. Amrane, M. Rafatullah, A. Hawari and M. A. Al-Ghouti, *Journal of Industrial & Engineering Chemistry*, 2014, **20**, 2939-2947.

10. Z. Chen, L. Cui, G. Zeng, D. Huang, C. Yang, W. Yang, Y. Zhou and C. Min, *Water Research*, 2016, **95**, 103-112.

11. M. Sevilla, P. Valle-Vigón, P. Tartaj and A. B. Fuertes, *Carbon*, 2009, **47**, 2519-2527.

12. P. Chingombe, B. Saha and R. J. Wakeman, *Journal of Colloid & Interface Science*, 2006, **302**, 408-416.

13. N. W. Brown, E. P. Roberts, A. Chasiotis, T. Cherdron and N. Sanghrajka, *Water Research*, 2004, **38**, 3067-3074.

14. I. D. Kovaios, C. A. Paraskeva and P. G. Koutsoukos, *Journal of Colloid & Interface Science*, 2011, **356**, 277-285.、

15. D. L. D. Lima, C. P. Silva, R. J. Schneider and V. I. Esteves, *Talanta*, 2011, **85**, 1494-1499.

16. J. Lu, Y. Li, X. Yan, B. Shi, D. Wang and H. Tang, *Colloids & Surfaces A Physicochemical & Engineering Aspects*, 2009, **347**, 90-96.

17. W. W. Tang, G. M. Zeng, J. L. Gong, Y. Liu, X. Y. Wang, Y. Y. Liu, Z. F. Liu, L. Chen, X. R. Zhang and D. Z. Tu, *Chemical Engineering Journal*, 2012, **s 211–212**, 470-478.

18. W. Zhang, J. Zheng, P. Zheng and R. Qiu, *Chemosphere*, 2015, **134**, 438-445.

19. A. Dutta and N. Singh, *Environmental Science and Pollution Research*, 2015, **22**, 3876-3885.

20. X. Ren, J. Li, X. Tan and X. Wang, *Dalton Transactions*, 2013, **42**, 5266-5274.

21. M. Imamoglu and O. Tekir, *Desalination*, 2008, **228**, 108-113.

22. M. M. Rao, A. Ramesh, G. P. Rao and K. Seshaiah, *Journal of Hazardous Materials*, 2006, **129**, 123-129.

23. R. Laus, T. G. Costa, B. Szpoganicz and V. T. Fávere, *Journal of Hazardous Materials*, 2010, **183**, 233-241.

24. E. Salehi, S. S. Madaeni, L. Rajabi, V. Vatanpour, A. A. Derakhshan, S. Zinadini, S. Ghorabi and H. A. Monfared, *Separation & Purification Technology*, 2012, **89**, 309-319.

25. C. S. Castro, M. C. Guerreiro, M. Gonçalves, L. C. Oliveira and A. S. Anastácio, *Journal of Hazardous Materials*, 2008, **164**, 609-614.

26. X. Zhao, W. Ouyang, F. Hao, C. Lin, F. Wang, S. Han and X. Geng, *Bioresource Technology*, 2013, **147**, 338-344.

1.  Corresponding author: Tel.: +86-731-88822778; Fax.: +86-731-88823701

   E-mail: [tanglin@hnu.edu.cn](mailto:tanglin@hnu.edu.cn) (L. Tang), [zgming@hnu.edu.cn](mailto:zgming@hnu.edu.cn) (G.M. Zeng). [↑](#footnote-ref-2)
2.  [↑](#footnote-ref-3)
